# Modeling Patient Flow in an Emergency Department under COVID-19 Pandemic Conditions: A Hybrid Modeling Approach

**DOI:** 10.3390/healthcare10050840

**Published:** 2022-05-02

**Authors:** Gaute Terning, Eric Christian Brun, Idriss El-Thalji

**Affiliations:** 1 Department of Safety, Economics, and Planning, University of Stavanger, 4036 Stavanger, Norway; eric.brun@uis.no; 2 Department of Mechanical and Structural Engineering and Materials Science, University of Stavanger, 4036 Stavanger, Norway; idriss.el-thalji@uis.no

**Keywords:** healthcare, emergency department, patient flow, simulation modeling, agent-based modeling, pandemic decision support

## Abstract

Emergency departments (EDs) had to considerably change their patient flow policies in the wake of the COVID-19 pandemic. Such changes affect patient crowding, waiting time, and other qualities related to patient care and experience. Field experiments, surveys, and simulation models can generally offer insights into patient flow under pandemic conditions. This paper provides a thorough and transparent account of the development of a multi-method simulation model that emulates actual patient flow in the emergency department under COVID-19 pandemic conditions. Additionally, a number of performance measures useful to practitioners are introduced. A conceptual model was extracted from the main stakeholders at the case hospital through incremental elaboration and turned into a computational model. Two agent types were mainly modeled: patient and rooms. The simulated behavior of patient flow was validated with real-world data (Smart Crowding) and was able to replicate actual behavior in terms of patient occupancy. In order to further the validity, the study recommends several phenomena to be studied and included in future simulation models such as more agents (medical doctors, nurses, beds), delays due to interactions with other departments in the hospital and treatment time changes at higher occupancies.

## 1. Introduction

Emergency departments (EDs) are complex and crucial systems for accommodating patients in urgent and responsive need of health care [1]. Lately, there has been a particular focus on the ED under COVID-19 pandemic conditions [2], forcing us to rethink its organization [3]. Due to the pandemic conditions, EDs have had to adjust their operations according to regulations while cost-effectively managing their resources.

In order to comply with restrictions and guidelines, several management policies have been imposed simultaneously, e.g., changes in patient arrival handling, priorities of patients with suspected virus contamination, structural changes in patient flow, and the use of available space. Although such measures have put unprecedented strain on the department and its vital resources, they aim to ensure less overcrowding and treatment time while keeping the risk of contamination as low as possible.

In the past, ED crowding has been shown to have adverse effects on patient response time [4,5,6,7,8]. Overcrowding and treatment time can be regarded as systemic effects of several agent behaviors and multiple operating scenarios, which might be hard to explain by medical staff or revealed by field experiments or surveys. Computer modeling and simulation, in particular agent-based modeling, can thus be useful, as it allows to model organizational participants with their collective behavior [1,9,10,11,12]. It can further be used for decision support and to evaluate interventions, scenarios, operational risks, and cost-effectiveness of policies.

Overall, there is an increasing body of literature on modeling and simulation of ED patient flow [13,14]. There are several simulation models where the discrete event or system dynamics approaches mimic ED patient flow during normal conditions [13]. Such modeling and simulation approaches have also been used to model COVID-19 spread and transmission [4] and mass-vaccination facilities [15]. However, there is an unmet need for simulation models that mimic, explain, and predict ED patient flow under pandemic conditions while considering multiple agent behaviors [11], a feature not available in the discrete event or system dynamics approaches. Moreover, there is a lack of studies demonstrating rigorous and transparent construction of a conceptual model of an ED, which leads to difficulties for readers to understand, assess and build trust in such models. Furthermore, from a practitioner’s point of view, there are several performance measures that ED managers use (such as time to treatment, the average length of stay, % full of triage room, etc.) that are not yet considered in existing simulation models.

This paper thus aims to answer the following research question:

*How can we build a simulation model of the emergency department patient flow during COVID-19 pandemic conditions while considering multiple agent behaviors?*

In this paper, we present a thorough description of how we developed such a simulation model. We first detail how we developed a conceptual model building on system knowledge and expertise from a case organization. Following that, we show how this conceptual model was incorporated into a hybrid computational model, where agent-based modeling was used to model patient behavior, and discrete event modeling was used to model resources (e.g., treatment rooms, triage beds, extra treatment rooms, pre-triage). The modeling approach we used consisted of (1) case study and systems analysis, (2) conceptual modeling, (3) computational modeling, and (4) verification and validation. Moreover, the main key performance indicators (KPIs) for patient flow used by practitioners were integrated and visualized.

The remainder of this article is organized as follows: the following Materials and Methods section will briefly illustrate the applied four-step simulation modeling process, fundamentals of the main methods used, description of the case study (process, facility), and input data (patient arrival data). Next, the Results section will provide rigorous documentation of the conceptual model, including the purpose, KPIs, interfaces, process flows, and sequential interactions between agents. Additionally, the computational model and a comparison between the simulated and real-world data will be presented. Finally, the Discussion section will go through the implications and possibilities drawn from the results, and the Conclusions section will state the final, conclusive remarks of the research findings.

## 2. Materials and Methods

This section will present the modeling methodology and empirical case. First, the simulation modeling methodology and methods to collect and analyze the model inputs and structure will be presented for both conceptual and computational models. Next, we will present our case organization—the ED of the Stavanger University Hospital in Norway—and the case data that were used to build, validate, and verify the models.

### 2.1. Simulation Modeling Methodology: Randers’ Model

**Figure 1:**
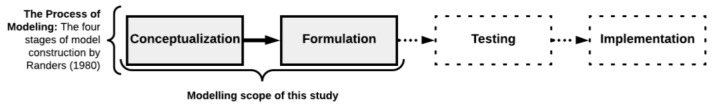
Randers’ modeling methodology.

In the original literature on modeling and simulation, there are several formalized ways of carrying out simulation studies [16]. In this study, we followed the process defined by Randers’ [17], which divides the process of modeling into four main phases: (1) conceptualization, (2) formulation, (3) testing, and (4) implementation (see Figure 1).

Randers’ structure was chosen because it focuses on model purpose rather than a singular and finite problem as a starting predicate for the model. More importantly, Randers’ approach is perhaps the most paradigm-independent among the available modeling protocols from the research literature. Therefore, in the context of a hybrid model which combines two or more paradigms (discrete-event and agent-based modeling) into one simulation study, Randers’ protocol was considered the most appropriate. The methodological aspects that will be given primary focus hereunder are those of the “conceptualization” and “formulation” steps. The last two steps were out of scope for this paper but will be pursued by the authors in a subsequent paper.

#### 2.1.1. Conceptual Modeling

In a previous paper [18], we developed and presented a general model for an ED, mimicking the patient flow process before the pandemic situation. In this study, the model has been expanded and presented in further detail to cover patient regimens during a pandemic situation. Like the general model, this model expansion was carried out in collaboration with the case organization through several meetings. As recommended in the literature, this stage was started before any computational modeling was made [19].

**Figure 2:**
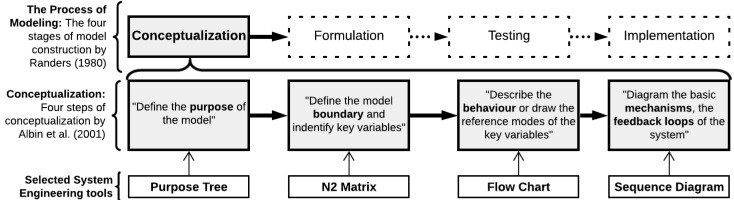
Model conceptualization process defined by Albin [20] and the associated systems engineering tools.

The conceptual modeling followed the four steps of Albin’s [20] process for constructing rigid conceptual models, shown in Figure 2. The four steps are: “Step 1–Define the purpose of the model”, “Step 2–Define the boundary and key variables”, “Step 3–Describe the system behavior”, and “Step 4–Describe the basic mechanisms of the system”. Although the steps here are numbered from 1 to 4, it does not imply a necessity for a strict chronological enactment. The overall simulation modeling process is a recursive and iterative process that will be developed in a back-and-forth manner, an issue we will revert to in Section 2.3.4.

For each step we used a system engineering tool to aid communication with the case organization and to help analyze and understand the processes together with the stakeholders. The steps and their corresponding tools are shown in Figure 2.

The following subsections describe these four systems engineering tools.

#### 2.1.2. Purpose Tree

**Figure 3:**
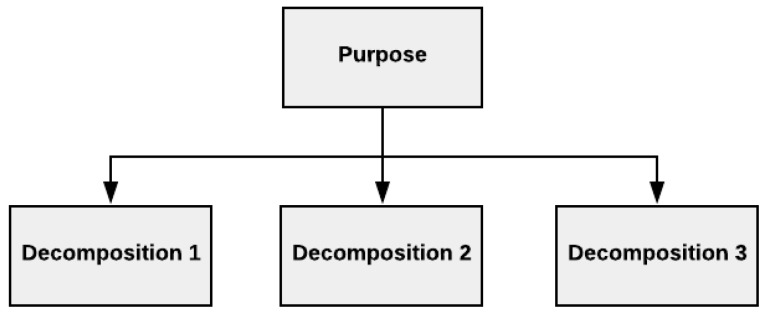
A generic purpose tree and its KPIs decomposition.

A purpose tree, shown in Figure 3, is an illustrative tool representing the purpose behind the simulation model of interest and its measurable key performance indicators (KPIs). Different versions of tree structures are commonly used in management disciplines, e.g., work breakdown structure in the project management discipline, and organization charts. The purpose tree is used similarly to elaborate the fundamental aim of the model and decompose it to achieve an overview of the model purpose and what system elements the model purpose builds upon.

#### 2.1.3. Interface Diagram

**Figure 4:**
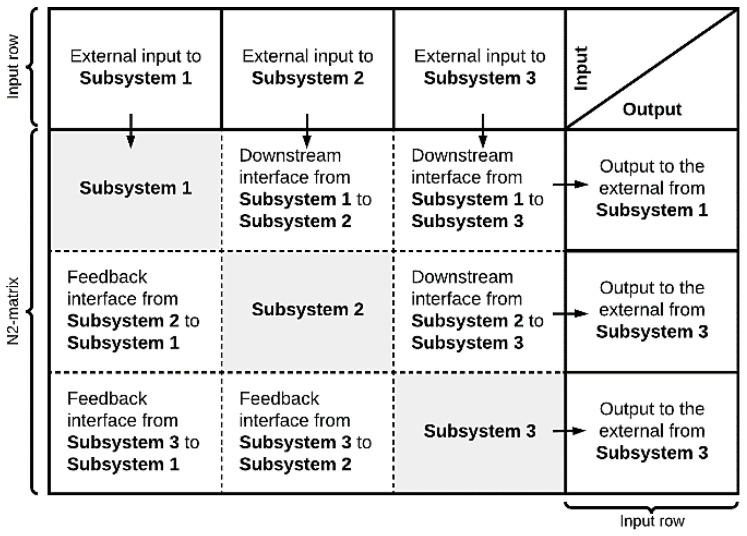
Generic interface diagram.

The interface diagram, shown in Figure 4, also known under the name “N^2^-matrix”, visually illustrates the interfaces between involved entities or agents, as well as external inputs. The principal diagonal (Principal diagonal: the diagonal going from the uppermost left corner, “Subssytem 1” in Figure 4, to the lowermost right corner, “Subssystem 3” in Figure 4.) in the matrix constitutes different subsystems. The remaining squares in the matrix constitute interfaces between the various subsystems. The blocks above the principal diagonal constitute downstream interfaces, and the blocks below the principal diagonal constitute interface feedback upstream in the system. The top row of the matrix contains descriptions of subsystem input from external parts. The rightmost column of the matrix illustrates the output from the subsystems.

The principal diagonal of the N^2^-matrix may be procedural steps of a process, or a distinct group of assets used by system agents. Figure 4 shows a generic interface diagram where subsystems 1–3 are considered. Like the purpose tree, this tool is flexible as it can be used at several different units (e.g., health, patient, medicine, information) and levels (e.g., health trust, hospital, department, ward, treatment room) of analysis. For multi-purpose models, one can construct one diagram for each purpose and analyze them separately.

#### 2.1.4. Flow Chart

**Figure 5:**
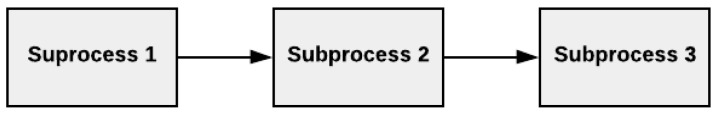
A generic flow chart of a process containing three linearly connected subprocesses.

A flow chart, shown in Figure 5, is a high-level description of the system’s behavior. It describes the process in terms of connected steps necessary to accomplish a task. In our study, the flow chart representation was divided into states that coincide with the most important and distinct processes the patient goes through in the ED.

#### 2.1.5. Sequence Diagram

**Figure 6:**
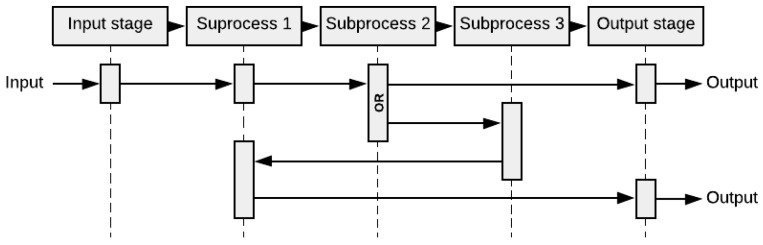
Simple illustration of a sequence diagram progressing through three subprocesses.

The sequence diagram, shown in Figure 6, is a detailed-level description that shows the entity/agent interactions arranged in a time sequence. Complex social systems, e.g., EDs, rarely have a linear progression through the system’s different subprocesses. The system may have several feedback loops, resulting in a plethora of different system realizations, depending on specific details of the agent traveling through the system.

The sequence diagram provides a systemic way to capture, analyze, and discuss how different system agents progress throughout the various subprocesses of the system. A fully detailed sequence diagram thus visualizes all the unique passages an agent can take through the system in a more detailed manner than a flow chart can illustrate.

### 2.2. Computational Modeling–Hybrid Simulation Modeling

**Figure 7:**
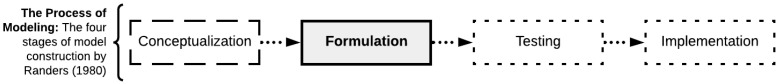
In the process of modeling, the next step after the conceptual modeling is formulation.

Following the conceptualization step, the next step in the overall modeling was to perform the Formulation step, as shown in Figure 7. The formulation is about structuring the conceptual model into software by using a computer programming language. A computational model will thus be another layer of codification on the conceptual model and be significantly contingent on the software’s codification abilities (i.e., a meta-model).

In order to carry out the modeling, it was found necessary to use an agent-based simulation in order to be able to adequately implement the complex patient flow logic of the case organization. However, a pure agent-based modeling approach was found to be insufficient when we were to model the resources and their use by the patient agents. Some agents would behave dominantly in a discrete-event manner, e.g., room seizing and releasing.

This required us to expand from strictly agent-based modeling (ABM) to a hybrid combination by including discrete event simulation (DES) model elements. To implement this type of hybrid model that allowed for the utilization of both ABM and DES, we used AnyLogic 8 Personal Learning Edition 8.7.2.

Agent behavior was defined using the statechart, which is an integrated feature in AnyLogic describing agent behavior. A statechart is a blueprint for the behavior of each agent. The statechart is common for every agent (of the same type) that becomes initiated into the model. However, every patient will each have their individual realization of the statechart. The resource allocation logic, which included elements of discrete-event modeling, was codified by using the “process modeling” flow chart in AnyLogic.

In making the model, we collected data about how the emergency department in our case study normally works in a pre-pandemic situation. This model served as a basis for the model development. We then added the complexities introduced by the pandemic restrictions, e.g., a waiting zone, extra treatment rooms, and the associated procedures.

We then validated the model in two manners: Firstly, we set the patient contamination rate (PCR) to 0% to simulate a pre-pandemic situation, ran the model with real-life pre-pandemic patient arrival data and compared the output of the run with real-life pre-pandemic patient crowding data.

Secondly, we ran the model with peri-pandemic patient arrival data, using a PCR set to 30%, since this was the actual patient contamination rate in the real peri-pandemic data, and again compared to the real-life peri-pandemic patient crowding data.

### 2.3. Case Study: ED of Stavanger University Hospital

In order to obtain a good understanding of the studied ED, three aspects will be presented in this section:

(1). The facility and layout to understand the capacity, routes, and waiting zones.
(2). The data management systems that store and visualize the patient arrival and crowding rates, (in this case, two systems called Meona and Smart Crowding, respectively).
(3). The involved experts who provided descriptions of operations, policies, and expected performance measures.

#### 2.3.1. Facility and Layout

The case subject of this study is the ED of the public hospital Stavanger University Hospital, located in the city of Stavanger in the south-western part of Norway. The hospital is a large hospital with more than 500 beds, over 7800 employees, and 33 wards serving 369,000 city inhabitants. The case ED serves approximately 35,000 patients each year, averaging around a hundred patients daily. During normal circumstances, the case ED is equipped with 13 treatment rooms, 11 triage beds, and seven medical doctors (1 foundation house officer, three surgeons, two neurologists, and one orthopedist) [21,22,23,24].

#### 2.3.2. Case Data

**Figure 8:**
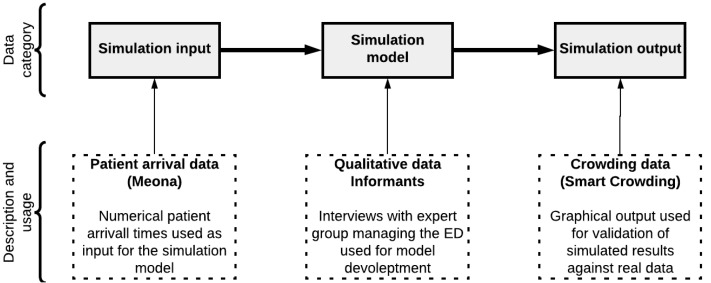
The data categories used in constructing, running, and validating the simulation model.

Three categories of data were utilized in this study, displayed in Figure 8; (1) data used as inputs to run the simulation model, (2) data used to construct the simulation model, and (3) data used to validate the simulation output. Figure 8 shows a schematic overview of the case data categories.

The first category—data used for simulation input—consisted of anonymized patient arrival time registrations for patients in the ED. These data were pre-existing data recorded independently of this study and were thus secondary data. The dataset we utilized was collected from a local database in an information and communication technology (ICT) system called Meona at the case hospital. Each record of data in Meona represented the time of arrival to the ED of one new patient. The number of data entries on a particular day in Meona thus represented the number of patients arriving at the ED that day.

The second category—data used for simulation model developments—was mainly obtained through interviews with knowledgeable stakeholders from the case organization. This category of data represented information about the patent flow process in the ED. These data were thus primary qualitative data. The steps in the patient flow process, and the criteria for the choice of alternative routes through the system, were explained to us by a group of key stakeholders at the case hospital.

**Figure 9:**
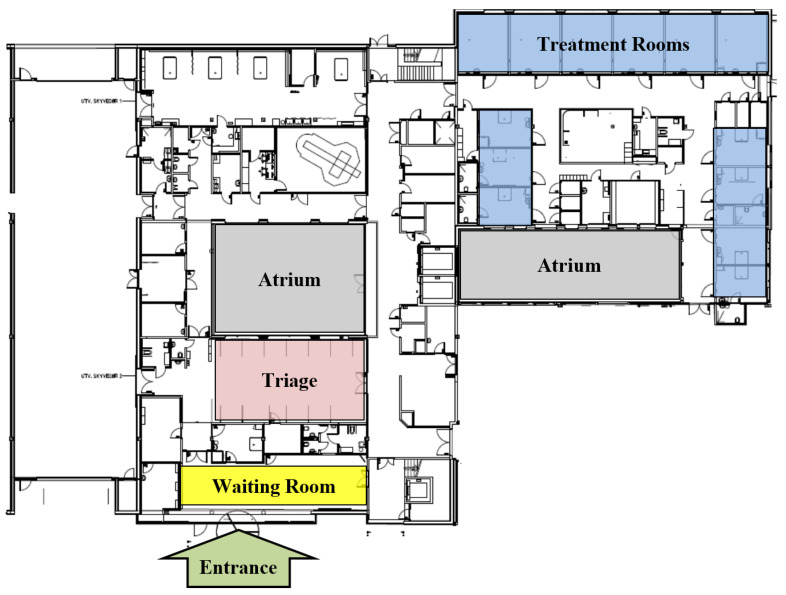
Blueprint of the studied ED before the pandemic measures was put in place.

Furthermore, we were given a blueprint of the layout of the ED, shown in Figure 9, which served as an outset for the construction of the computational model.

Using the blueprint eased the process of mapping out the patient movement path and laying out the resources. In addition to being informative in the model layout, this was also an important part of data collection to understand how the ED operates. For example, knowing the spatial positioning of the resources was necessary for ensuring that the model represented the actual system. Additionally, it directly revealed how many resources there were, e.g., number of treatment rooms which were limited to 13, and amount of triage beds limited to 11.

Additionally, we were given a walkthrough inside the case ED to observe the daily operation and obtain an understanding the workings of the real system.

For the final category—data used for the simulation output—we used secondary data retrieved from an ICT system at the hospital called SmartCrowding for verification and validation. These data consisted of graphs showing the patient flow development throughout the time of the days. Patient flow development was represented through a number of measures, such as time of day when crowing reached a certain percentage of room capacity, peak crowding level, etc. Thus, while the data from Meona represented the actual arrival of patients to the ED on a particular day, the data from SmartCrowding showed the actual resulting patient flow development on the same day.

#### 2.3.3. Arrival and Crowding

The set of real-life numerical data we were given, gathered from Meona, consisted of registration times for each patient’s arrival at the ED. The record of the data spanned over two full days, altogether 48 h. Our informants selected these two days as two representative days. These two days of data collection were two regular separate working days at the hospital (i.e., *not in sequence*) in the *pre-pandemic* situation, i.e., *prior* to the COVID-19 pandemic onset. The crowding data for the same two days, gathered from SmartCrowding, are shown in Table 1a.

We were also given a similar set of real-life numerical data gathered from Meona during the pandemic, i.e., in a peri-pandemic situation, which consisted of registration times for patients arriving at the ED in the case hospital. Data spanning over two separate full days were selected also from this dataset. The real-life crowding data for the same two days, gathered from SmartCrowding, are shown in Table 1b.

#### 2.3.4. Operations and Experts’ Descriptions

Dialog with a case organization is essential for a modeling process to succeed and benefit the system stakeholders. Accordingly, a significant portion of the data for this study was the qualitative data gathered through talking with the organization’s stakeholders. These individuals are personnel that work closely with the ED on a day-to-day basis.

The communication occurred as a mix of meetings on both a scheduled basis and an on-need basis for clarification in the model development. Development of both the conceptual and the computational model required intensive gathering of qualitative data by direct communication with the case organization. Testing of the computational model would quickly reveal misunderstandings in the conceptual modeling, resulting in an iterative development process in dialog with the stakeholders, as we indicated in Section 2.1.1. The development process, shown in the overall modeling methodology in Figure 2, was, thus, in practice, a nonlinear process, where the steps had to be revisited continually and iteratively throughout the lifetime of the model’s development process.

## 3. Results

Applying Randers’ model of simulation modeling [17] allowed us to obtain three main results:

(1) The conceptual model that represents the real-world structure and context of the ED during both pre-pandemic and peri-pandemic status.
(2) The computational model that mimics the patient flow behavior in the ED during both the pre-pandemic and peri-pandemic situations.
(3) The output (the simulated behaviors) of the computational model, where both real (from the patient crowding data) and simulated behavior are compared.

### 3.1. Result from Conceptual Modeling of the Case

Performing the conceptual modeling process described in Section 2.1.1 and Figure 2, resulted in a model including the following elements:

(1) Definition of the model purpose and twelve performance measures that the simulation model should be able to perform.
(2) Definition of model boundary and key variables, including critical interfaces between the *layout agents* of the ED (pre-treatment, triage, treatment rooms, waiting-zones, discharge).
(3) Description of the system behavior, including interactions between *patient agents* (ordinary and contaminated patient) and *layout agents* to describe the room seizing/releasing process, and
(4) Description of the basic mechanisms of the system.

#### 3.1.1. Step 1—Definition of the Purpose of the Model

**Figure 10:**
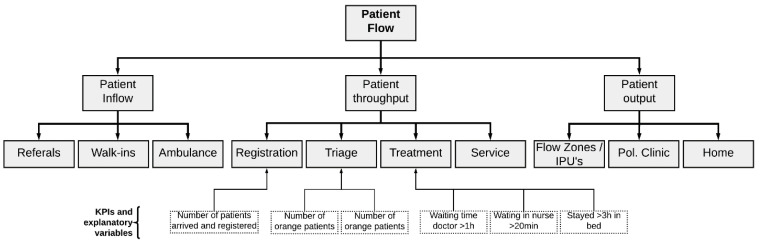
Purpose tree breakdown structure and KPIs for ED operations.

The model’s primary purpose was to model the patient flow behavior in the ED to test several policies and interventions in a virtual and low-cost environment. From an operational perspective, the patient flow could be divided into three main segments, as illustrated in Figure 10: patient inflow, patient throughput within the department, and patient output.

##### Implementation of the Patient Flow Key Performance Indicators

Performance indicators were selected and constructed within the simulation software to measure relevant aspects of patient flow within the ED. The following comprises a brief description of the KPI implementation as well as their mathematical equations (main contributor of this paper is the author of these equations), illustrating the calculations programmed into the simulation model.

1. Time to treatment (TTT) [h/pt]: This KPI tracks the time spent on all the activities prior to the treatment. Then, it is calculated as an average. TTT is calculated for all the patients (Tot.), the patients that are suspected to be pathogen contagious (Cont.), and the patients that are found likely not to be pathogen carrying (Ord.).

$$\mathrm{TTT}\left(t\right)=\frac{1}{N_{\mathrm{out}}\left(t\right)}\cdot \sum_{n\;=0}^{N_{\mathrm{out}}\left(t\right)}\left(P_{\mathrm{Time}\;\mathrm{entering}\;\mathrm{TR}}\left(n\right)-P_{\mathrm{Time}\;\mathrm{entering}\;\mathrm{ED}}\left(n\right)\right)\;\forall \;P\in \left[P\;\left(0\right),\;\ldots ,\;P\left(N_{\mathrm{out}}\left(t\right)\right)\right]$$

$P\left(n\right)$–*n*th patient agent, $P_{x}\left(n\right)$–parameter *x* of the nth patient agent, $N_{in}\left(t\right)$–Number of patients who entered the ED at time *t*,$\;N_{out}\left(t\right)$–Number of patients left the ED at time *t*, t–time; acting as the independent discrete-time variable going from 00:00:00 to 23:59:59, running in increments of seconds.

2. The average length of stay (ALOS) [h/pt]: This calculates the average time the patients spend in the ED. This measure can also be calculated individually for the different patient populations.

$$\mathrm{ALOS}\left(t\right)=\frac{1}{N_{\mathrm{out}}\left(t\right)}\cdot \sum_{n\;=0}^{N_{\mathrm{out}}\left(t\right)}\left(P_{\mathrm{Time}\;\mathrm{leaving}\;\mathrm{ED}}\left(n\right)-P_{\mathrm{Time}\;\mathrm{entering}\;\mathrm{ED}}\left(n\right)\right)\;\forall \;P\in \left[P\;\left(0\right),\;\ldots ,\;P\left(N_{\mathrm{out}}\left(t\right)\right)\right]$$

$P\left(n\right)$–*n*th patient agent, $P_{x}\left(n\right)$–parameter *x* of the nth patient agent, $N_{in}\left(t\right)$–Number of patients who entered the ED at time *t*,$\;N_{out}\left(t\right)$–Number of patients left the ED at time *t*, t–time; acting as the independent discrete-time variable going from 00:00:00 to 23:59:59, running in increments of seconds.

3. Crowding [%]: Crowding is defined as the number of patients simultaneously staying within the ED facility, i.e., prevalence. This measure tracks how long the crowding in the ED is above certain predefined levels and divides it by the total amount of time passed. The crowding levels are selected according to the case organization’s plan for high activity, 15, 20, and 30.

$${\mathrm{Crowding}}_{>15}\left(t\right)=\frac{\sum_{n\;=0}^{t}u\left(t\right)}{t}\;\cdot 100\%,\;\;\;\;\;u\left(t\right)=\left\{\begin{array}{c} 1\;\mathrm{if}\;\left(N_{\mathrm{out}}\left(t\right)-N_{\mathrm{in}}\left(t\right)\right)>15\\ 0\;\mathrm{otherwise} \end{array}\right.$$

$P\left(n\right)$–*n*th patient agent, $P_{x}\left(n\right)$–parameter *x* of the nth patient agent, $N_{in}\left(t\right)$–Number of patients who entered the ED at time *t*,$\;N_{out}\left(t\right)$–Number of patients left the ED at time *t*, t–time; acting as the independent discrete-time variable going from 00:00:00 to 23:59:59, running in increments of seconds.

4. Peak crowding: This measure is intended to present information on how many patients are present at the peak of the day.

$$Peak\;crowding\left(t\right)=max\left[N_{out\;}\left(t\right)-N_{in}\left(t\right)\right]\;\forall \;\;t\in \left[0,\;t\right]$$

$P\left(n\right)$–*n*th patient agent, $P_{x}\left(n\right)$–parameter *x* of the nth patient agent, $N_{in}\left(t\right)$–Number of patients who entered the ED at time *t*,$\;N_{out}\left(t\right)$–Number of patients left the ED at time *t*, t–time; acting as the independent discrete-time variable going from 00:00:00 to 23:59:59, running in increments of seconds.

5. Time of peak [time]: This measure states records at which the previously mentioned peak of crowding occurs.
6. Time start use [time]: This measure keeps track of when the different resources start being used within the ED. Measures were chosen to be tracking the use of the extra treatment rooms (*E.Tr.*), triage (*Tri.*), and the waiting zone (WZ).
7. Time in use [%]. This estimates the amount of time the resources are used as a proportion of the total time simulated. Equation (5) shows the calculation for the extra treatment rooms (*E.Tr.*).

$$Time\;in\;use_{E.Tr.}\left(t\right)=\frac{\sum_{n=0}^{t}u\left(t\right)}{t}\;\cdot 100\%,\;\;\;\;\;u\left(t\right)=\left\{\begin{array}{c} 1\;\mathrm{if}\;N_{@E.Tr.}\left(t\right)>0\\ 0\;\mathrm{otherwise} \end{array}\right.$$

$P\left(n\right)$–*n*th patient agent, $P_{x}\left(n\right)$–parameter *x* of the nth patient agent, $N_{in}\left(t\right)$–Number of patients who entered the ED at time *t*,$\;N_{out}\left(t\right)$–Number of patients left the ED at time *t*, t–time; acting as the independent discrete-time variable going from 00:00:00 to 23:59:59, running in increments of seconds.

8. Time full [time]: Similar to the “time start use”, this measure keeps track of when the resource first reached its full capacity (i.e., there was no more left of the resource for a new patient for the first time).
9. Waiting time pr pts [h/pt]: This measure calculates the average waiting time in WR across the different patient agent groups.
10. Times TR blocked for contaminated patients [#]: This measure keeps track of the number of times a patient with virus suspicion is blocked from going directly to a treatment room after undergoing the pre-triage screening. Such a blockage occurs under the following conditions: (1) all the ordinary and all the extra treatment rooms are fully utilized, and (2) no ordinary patients can leave their treatment room, either due to the lack of a waiting zone available or because none of the patients currently in the treatment rooms have stayed their minimum amount of time in their treatment rooms. Therefore, this measure is critical, because if this condition occurs, it means that a potentially contaminated patient has to wait, which poses an increased risk of contamination.
11. Times TR (WZ) seized [#]: Similar to the previous, this one is a pure counter. This measure counts the number of times a treatment room (or waiting zone) is seized by a patient agent. The main reason for keeping track of this is that seizing rooms is work intensive. Rooms need to be set up and sanitized and thus constitute an economic and resource burden both directly and indirectly. Additionally, besides the pure labor aspect, the management of seizing and releasing may cause an error and be costly. In addition, having patients leave their rooms might be a high cost for the individual patients, as this might yield stress and other discomforts for the patient.

#### 3.1.2. Step 2—Definition of Model Boundary and Key Variables

The organizational structures of EDs may vary across hospitals. In the case organization, the ED was organized as its own independent department, not as a subdivision of another department, which is typical in smaller Norwegian hospitals.

**Figure 11:**
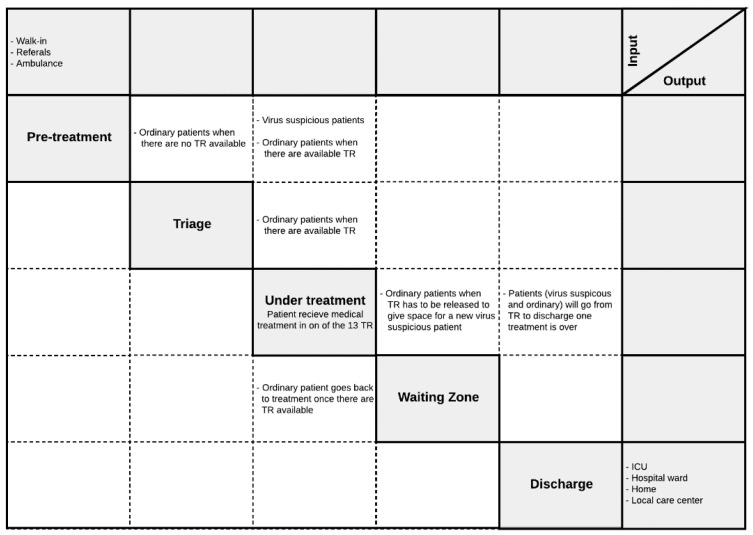
N2 diagram for ED facilities under pre- and peri-pandemic conditions.

In this step, the overall purpose was to convey patient flow elements in the simulation model. Figure 11 illustrates the inputs and outputs of each process step, identified by applying to our case the Interface (N2) diagram tool presented in Section 2.1.3.

#### 3.1.3. Step 3—Description of the System Behavior

**Figure 12:**
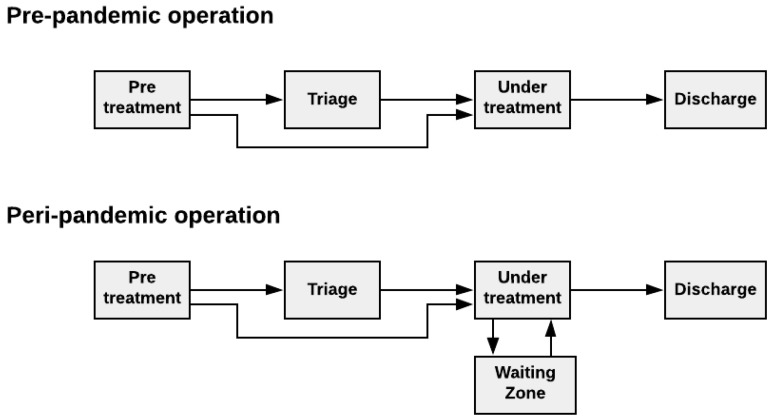
Flow chart for patient flow under the pre-and peri-pandemic situation.

In this step, a simple flowchart depiction of the patient flow process was developed. The result is shown in Figure 12. The following descriptions explain each element in the flowchart:

##### “Pre-Treatment”

The first stage in the flow chart was pre-treatment. This stage included everything from when the patient arrived at the ED until the patient left registration to be admitted into either the triage or treatment room. Imposed by the pandemic situation, the ED had decided to install a pre-triage area outside the entrance of the ED, where all patients arriving at the ED needed to be pre-screened. From here, if it was found likely that the patient was infected by the COVID-19 virus, the patient would immediately be directed to a prepared treatment room (TR) to ensure a reduction in intra-departmental contamination. This implied that a treatment room was de-sanitized whenever it had been used to treat a patient who was suspected to have COVID-19.

##### “Triage”

Triage here refers to the physical location where admitted patients wait for treatment. It is used in situations where no treatment rooms are vacant. We will later address the distinction between action and physical location, as triage can refer to both the action of triaging a patient according to a specific triage system and the physical location within the ED where the triaging of patients is primarily taking place.

##### “Under Treatment”

This stage of the patient pathway is where the actual treatment takes place. Then, according to the prescribed medical protocol, the patient receives medical treatment from doctors and nurses. The standard treatment rooms in use before the pandemic amounted to 13. Extra treatment rooms were introduced for expanded capacity to cope with the pandemic situation. Once the standard treatment rooms were filled, the extra treatment rooms would be used to accommodate more patients.

To cope with the risk of transmission between the patients, patients suspected of being contaminated would be expedited to a treatment room. After treatment, the treatment room would be de-sanitized before accepting a new patient. If the regular treatment rooms were full and a new patient with suspected contamination arrived, then a patient that had stayed in the room for at least 1 h would have to leave their treatment room and move over to a waiting zone.

##### “Waiting Zone”

As said in the previous stage, the waiting zone was for patients evaluated not to be incumbents of the virus. Here, patients would wait until there was an available treatment room that they could return to for their treatment to continue. 

##### “Discharge”

The last stage in the flow chart was the discharge. This step is where the patient finally has undergone the treatment and is ready to be discharged from the ED.

#### 3.1.4. Step 4—Description of the Basic Mechanisms of the System

**Figure 13:**
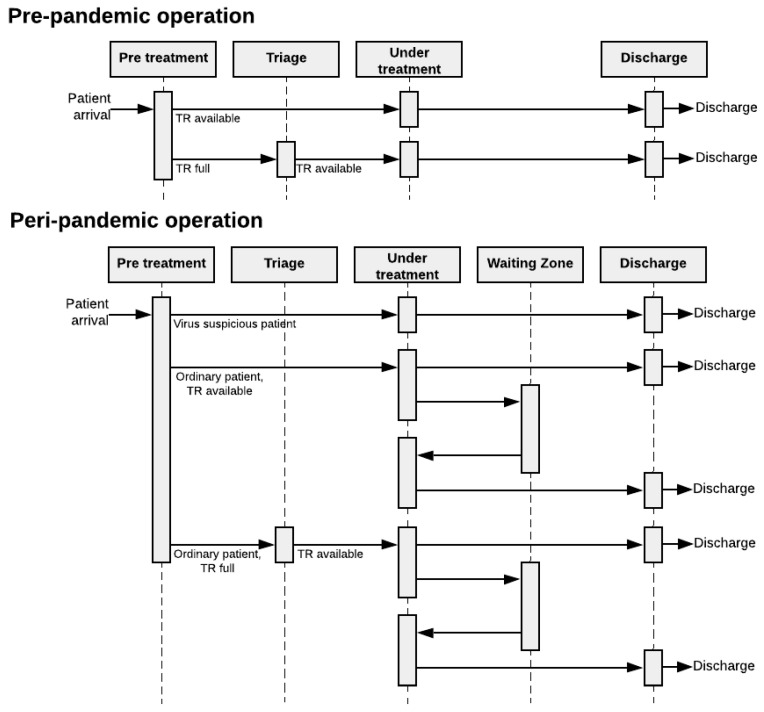
Effect on patient flow operation illustrated in sequence diagram pre- and peri-pandemic situation.

The fourth step in the development procedure was to capture the basic mechanisms of the system at a more detailed level than the simple flowchart. Discussions with the stakeholders in the case organization revealed a variety of possible pathways through the ED. These are illustrated in the sequence diagram in Figure 13, where the conditions for each route are indicated. These will be further elaborated in Table 2 in our computational model.

**Table 2:** Description of the elements (states, transitions and initial conditions) of the patient agent statechart.

Name	Description	Logic	Code
	Patient arrival will be arriving according to the numerical data attained from the case organization. Arrivals can be programmed based on a probability distribution that will make for a stochastical model. Alternatively, arrivals can be programmed according to recorded arrival times; the model will then be deterministic.		
**‘Stay’**	State-Stay: The sole purpose of this state-block is to calculate the length of stay of patients throughout their lifetime, i.e., the entire statechart. Timer3 here does the counting. No other logic is contained within this state block.	Timeout for each second in simulation in order to increment the timer value within Timer3 according to criteria in the code. Variables: (integer) v_LOS	Action: v_LOS += inState(Stay) ? 1: 0;
**‘S1’**	State-Pre-treatment: First compound state in the overall patient flow. This state contains all the states for the pre-treatment activities a patient is undergoing. Like the previous state, counting is performed. It is worth noting counter is placed here instead of the ‘Stay’ state block to reduce the calculation power needed for each discrete increment.	Timeout for each second in simulation in order to increment the timer value within Timer1 according to code. Variables: v_time_in_PreTriage, v_time_in_WR	Action: v_time_in_PreTriage += inState(PreTriage)? 1: 0; v_time_in_WR += inState(WaitingInWR) ? 1: 0;
**‘s1-1’**	State-Incidence: Patient agent takes place into the statechart after being transferred from the ‘Exit’ block in DES (Figure 14). The sole purpose of this state block is that t serves the programmatic purpose of letting the patient agent enter the statechart. Once the patient agent is instantiated into this state, it is immediately passed onto the transition.	-	-
**‘t1-1’**	Transition: Patient agent arrives from the DES. Transition is triggered on the string message “occurrence,” which is sent from the ‘Exit’-block in the DES model.	-	-
**‘s1-2’**	State–PreTriage: Patient agent is getting moved from the entry node to the Pre-triage node, i.e., light green field in Figure 14.	The patient agent initiates movement from the Entry node to the te pre triage node.	Action: moveTo(main.PreTriageNode);
**‘t1-2’**	Transition: Transition from the state PreTriage to branch B1.	Transition is executed periodically for every fixed time interval in order to ensure. Parameter used: main.p_RegistrationCheckInterval	
**‘B1’**	Branch: This branch carries out the selection for what path the patient agent should proceed in.	Three different outgoing transitions: (1) If there is a suspicion that the patient is contaminated, the patient will go to S2. (2) If the patient agent is found not to be contaminated, the patient will go to the waiting room after a specified waiting time, i.e., state s1-3. (3) T3: If the patient agent has virus suspicion and there are no more treatment rooms, the patient must wait in the Pre-triage.	(1) Condition: p_contaminated && !f_is_TR_full() (1) Action: main.enter_SeizeTR.take(this); (2) Condition: !p_contaminated && (v_time_in_PreTriage > main.p_minTimeInPreTriage) (3) Action: if (p_contaminated && f_is_TR_full() && v_BlockFlag == false) { main.v_Virus_Patient_TR_Decline ++; v_BlockFlag = true; }
**‘s1-3’**	State–WalkingToWR: Patient agent walking to the waiting room from the pre triage.	The patient agent walks along the path between the pre-triage and waiting room shown in Figure 14.	Action: moveTo(main.waitingRoomNode);
**‘t1-3’**	Transition: The patient agent is simply transitioning between walking to the waiting room and waiting in the waiting room.	This transition is triggered once the patient agent stops walking and arrives at its place in the waiting room.	-
**‘s1-4’**	State–WaitingInWR: This state for the patient agents when waiting in the waiting room (WR); see the node in Figure 14.	-	-
**‘t1-4’**	Transition: Here, the patient agent will attempt to see if it is ready to proceed.	This transition will trigger periodically. It will check if it can fulfill any of the transitions branch B4 for each periodic interval.	-
**‘B4’**	Branch: This is the pathway forward after the PreTreatment state. Patient agents from here either have to go to the treatment room, triage or wait further if the above-mentioned is full.	Three outgoing transitions: (1) T1–Going to triage if there are no more treatment rooms left, (2) T2–Going to a treatment room, and (3) Stay in the waiting room if the above-mentioned transitions do not fulfill their execution conditions. None of the transitions have dependencies on the patient virus suspicious status, as these are already sorted in the previous branch B1.	(1) Conditions: f_is_TR_full() && !f_is_Triage_full() && (v_time_in_WR > main.p_minTimeInWR) (1) Action: main.enter_seize_Triage.take(this); (2) Action: main.enter_SeizeTR.take(this); (3) Conditions: !f_is_TR_full() && f_is_WZ_empty() && f_is_Triage_empty() && (v_time_in_WR >main.p_minTimeInWR)
**‘S2’**	State–IntraTreatment: This is the second major compound state of the overall patient flow. As the name suggests, this is where the patient finally undergoes treatment. The patient agent is released from the treatment room seizing pathway shown in the DES block diagram (Figure 14)	The treatment is simulated by the patient agent waiting inside the treatment room. The treatment time differs according to whether the patient has suspicion of virus contamination. (1) Timer variable: v_time_in_TR. (2) Counter variable: main.p_number_room_exit	v_time_in_TR += inState(Treatment) ? 1: 0; main.p_number_room_exit++
**‘s2-1’**	State–WalkingToAndSeizingTR: This is the state the patient agent is contained within until it has been transferred from the seize-path, as mentioned earlier.	-	-
**‘t2-1’**	Transition–The patient agent is transitioned to the next state once the message is received from the	The patient agent is transitioned to the next state once the message is received from the	-
**‘s2-2’**	State–Treatment: This is the state in which the patient agent is under treatment.	The patient agent stays in this state while the counter is increasing.	-
**‘t2-2’**	Transition–Transition out of treatment leads to a branch where there are three options possible.	This transition is cyclical and repeats every second during model runtime for patients’ agents that stay within the treatment state.	-
**‘B2’**	Branch: This branch leads the patient agent to go to the waiting zone, leave the ED, or stay to continue the treatment.	The branch is leading to three outgoing transitions (1) T6: Patient agents pause the treatment and makes the patient go to the waiting zone. (2) T7: Patient agent has completed treatment and will head to exit the ED. (3) Treatment is not carried out, and it will proceed until it is either completed or has to pause because the treatment room needs to be seized by a patient suspicious of contamination.	(1) Action: main.enter_seize_WZ.take(this); (1) Condition: !p_contaminated && f_is_TR_full() && !f_is_WZ_full() && v_time_in_TR > main.p_minTimeTR && f_is_this_longest_in_TR(this) && f_is_any_contaminated_in_WR() && main.b_is_WZ_in_use (2) Action: main.enter.take(this); (2) Condition: p_contaminated ? v_time_in_TR > main.p_TreatmentTime_VirusSuspicion: v_time_in_TR > main.p_TreatmentTime_OrdinaryPatient
**‘S3’**	State–Triage: Compound state element emulating the patient standing by in the triage until there is room for the patient in the treatment rooms.	Time is tracked. Variable: v_time_in_Triage	v_time_in_Triage += inState(Triage) ? 1: 0;
**‘s3-1’**	State-WalkingToTriage: The patient agent walks from the waiting room node to the triage node. The patient agent is retrieved from the DES path shown in Figure 14, distributing the triage bed to the patient agent.	-	-
**‘t3-1’**	Transition–Transition is executed once the patient agent is transferred from the DES flow chart.	-	-
**‘s3-2’**	State–WaitingInTriage:	This state emulates the waiting time in the triage.	-
**‘t3-2’**	Transition: Transition for checking if it is time to leave the triage. Decision-making through the branch (B5) in the next row.	Transition executed periodically; every second patient stays state ‘WaitingInTriage.’	-
**‘B5’**	Branch: Checking if the patient agent can leave the triage to go to a treatment room.	The branch is leading to two outgoing transitions. (1) Transition for when there is a treatment room ready. (2) No treatment room is available for the patient agent, returning to the previous state.	(1) Condition: !f_is_TR_full() && f_is_WZ_empty() && !f_is_any_contaminated_in_WR() && (v_time_in_Triage > main.p_minTimeInTriage) (1) Action: main.enter_SeizeTR.take(this);
**‘S4’**	State-WaitingZone: This is the compound state encompassing the states that emulate the waiting zone for the patient agents.	This compound statement has no time counters as the time is directly relevant for when a patient agent will leave this state. However, as seen in the outgoing transition (T5), the patient agent can leave once there is room again for the patient agent to return to the treatment room.	-
**‘s4-1’**	State–GoingToWZ: The patient is retrieved from the DES chart, it has been allocated to a waiting zone, and walking to the spot it has been granted.	-	main.enter_seize_WZ.take(this);
**‘t4-1’**	Transition: Patient agent is finished walking to the waiting zone and will proceed by waiting in the waiting zone.	This transition is carried out once the patient agent has arrived at the granted waiting zone spot. This transition is executed by receiving a message from the DES-chart	-
**‘s4-2’**	State-WaitingInWZ: The patient agent is waiting in the waiting zone.	-	-
**‘t4-2’**	Transition: Transition going out from waiting in the waiting zone.	This transition is periodically executed every second of runtime.	-
**‘B3’**	Branch: Branch for going further to the treatment room or keep on waiting.	This branch does have two outgoing transitions for the patient agents staying in the triage (1) T4–Going to the IntraTreatment state. (2) Keep on waiting in the triage.	(1) Condition: !f_is_TR_full() && f_is_WZ_empty() && !f_is_any_contaminated_in_WR() && (v_time_in_Triage > main.p_minTimeInTriage) (1) Action: main.enter_SeizeTR.take(this);
**‘S5’**	State–Discharge: Patient agent is here on its way out of the model. The state does not do anything besides being a mediator between the two states.	-	-

### 3.2. Computational Model

The following documentation of the computational model will be segmented into the following subjects: Environment, Resources, Agents, and Interaction topology, which are some of the elements STRESS guidelines suggested for documenting DES and ABS models [25].

The computational modeling process resulted in:

(1) Animated layout to visualize the patient flow behavior in a virtual ED environment.
(2) A discrete-event model for facility resources (treatment rooms, waiting zones, triage) and patient arrival rate.
(3) An agent-based model for patient flow (for ordinary and COVID-19 contaminated patients), where the sequence (state and transitions), policies (conditions, triggers), and service time (treatment time, waiting time) are modeled.

These will be presented in the following, and the modeling assumptions will be listed.

#### 3.2.1. Virtual ED Model

The computational model was modeled using AnyLogic 8.0 Learning Edition, and the present study was limited to considering the spatial resources of the ED. These resources were overlaid on top of the case organization’s blueprints for convenience and accuracy.

#### 3.2.2. Resource Allocation Model

The resource allocation of this model was programmed through a process flow chart using the discrete-event modeling approach.

#### 3.2.3. Patient Flow Behavior Model

The agents, depending on variable characteristics, will progress through the following main groups of activities:

- Pre-treatment;
- Triage;
- Intra treatment;
- Waiting Zone;
- Discharge.

**Figure 15:**
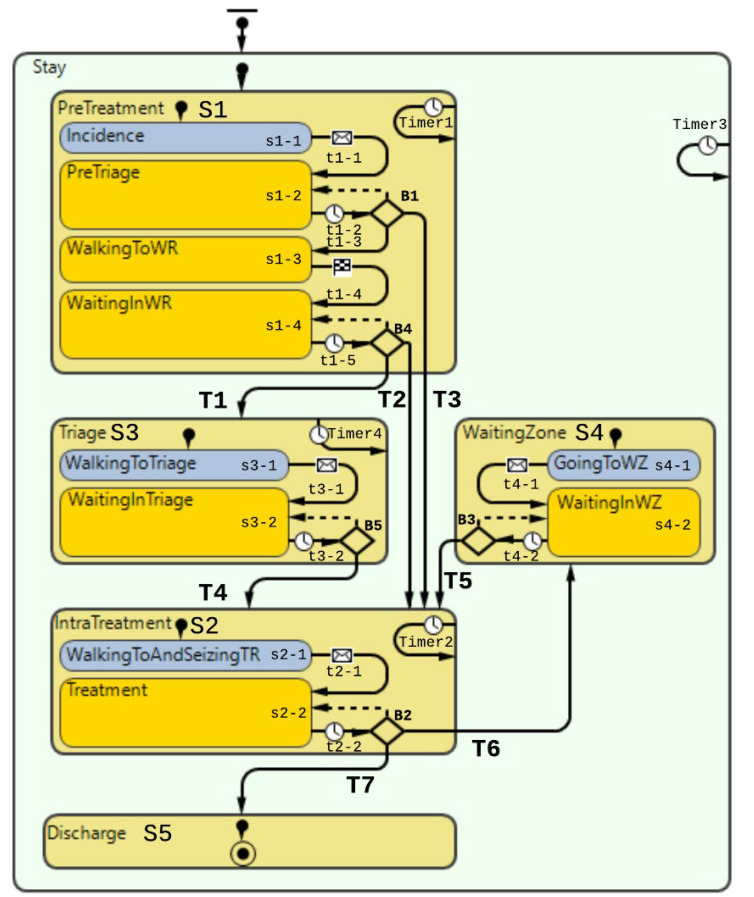
Statechart of patient flow behavior at the studied ED.

In Figure 15, the patient flow sequence is described in a statechart, where the main states (physical locations, e.g., triage, treatment room, waiting zones) and the triggers to move from one state to another are illustrated. The statechart is the programmatic entity that orchestrates the behavior of the individual agents in the model [26].

In our current model, the only agents acting are patients. Whether the patients are ordinary, or COVID-19 contaminated, patients have a clear flow throughout several facilities (triage, treatment rooms, waiting zones). Specific conditions trigger the flow or patient movement, e.g., if a treatment room is empty, a patient moves from triage into that treatment room. Thus, there are several states and transitions that each patient has to undergo within the ED. As there are specific transitions that are time-dependent and executed depending on how much time the patient agents have spent in different states, it was necessary to implement timers to keep track of the duration patients stay in different parts of the patient flow process.

As seen in the statechart (Figure 15), there is a correlation between the flowchart in the conceptual model developed in the previous subsections (Figure 12) and the resulting statechart. The main blocks in the flowchart and statechart are the same. However, the statechart has one more level of granularity as some of the main blocks (“PreTreatment”, “Triage”, “IntraTreatment”, “WaitingZone”) have a flow chart of activities within them.

In Figure 15, there is a unique transition (T3) for COVID-19 contaminated patients to move from the pre-triage room (S1-2) at B1 to treatment rooms (S2). This particular transition represents the mentioned priority rule that our case organization implemented in the ED during the pandemic for channeling the patients directly to treatment rooms.

In Table 2, the first three columns describe each state and transition and their logic in the following table. These were all discussed and verified by our stakeholders in the case organization. The rightmost column shows the code of each element implemented in the computational model.

### 3.3. Model Outcome and Output Validation

There are two primary outcomes from the computational model:

(1) Patient crowding timeline in the ED;
(2) Key performance indicators of patient flow, e.g., waiting time and time to treatment.

**Figure 16:**
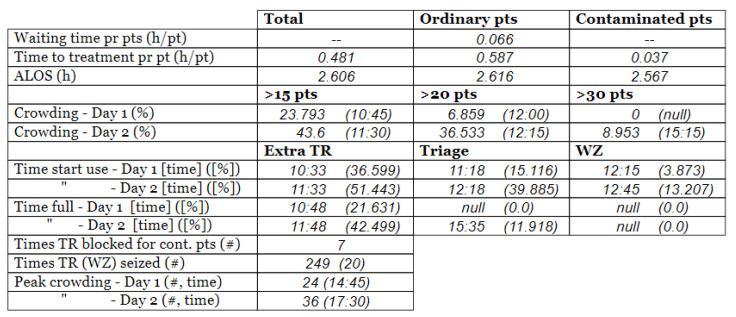
Snapshot of simulated KPI of patient flow in a pre-pandemic setting.

**Figure 17:**
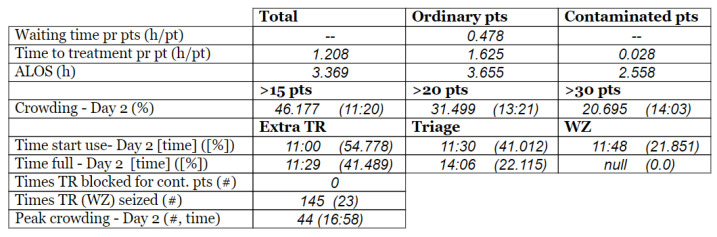
Snapshot of simulated KPIs of patient flow in a peri-pandemic setting.

Table 3 show comparisons between simulation output and actual patient crowding data in a pre-pandemic and peri-pandemic setting, respectively. Figure 16 and Figure 17 show snapshots of simulated key performance indicators during the same pre-pandemic and peri-pandemic settings, respectively.

As can be seen in Table 3, the curve shapes from the actual patient crowding data in the case organization system (SmartCrowding) in row (a) and our simulated patient crowding in row (b) show a reasonably close resemblance. There are, however, discrepancies of the primary two main types. Firstly, the curves from the actual data (row a) are more elevated than the simulation output curves (row b) and secondly, a slight time lag seems present between the actual curves and the simulated output curves. These discrepancies will be further discussed in Section 4.2.1 of this paper’s Discussion chapter.

In the conceptualization stage, we presented the key performance indicators of patient flow to be implemented and estimated by the simulation model. Figure 16 shows how these measures were represented in a table within the simulation software. The values will change dynamically throughout the simulation runtime.

As shown in Table 3, the curve shapes from the actual patient crowding data in row (a) and simulated patient crowding in row (b) also show reasonably close resemblance in a peri-pandemic setting. The same discrepancies between the curves for simulated and actual patient crowding (i.e., slight differences in elevation level and time lag) discussed above for Table 3 can also be observed here.

Figure 17 shows how key performance indicators were represented in the peri-pandemic setting. As in the pre-pandemic setting, the values will change dynamically throughout the simulation runtime.

## 4. Discussion

In this section, the conceptual and computational modeling process is discussed, followed by a discussion of the simulated results and their validity. Finally, some implications and future work will be highlighted.

### 4.1. Discussion of the Conceptual Modeling

The conceptual model in this present paper is documented in a paradigm-independent manner, providing freedom for modelers. Additionally, this approach provides transparency of the fundamental logic of the model. It makes it accessible for researchers determined to use a simulation paradigm or a mix of simulation paradigms different from the one used in this study. Furthermore, using systems engineering methods for conceptual modeling supports several purposes.

The purpose tree provided a direction for modeling by identifying and decomposing the over-arching purpose of the model. It also helped establish what key variables were essential to communicate in any output dashboard of the end product. The interface diagram aided us in mapping out both downstream and upstream patient flow interfaces between subcomponents of the system. Thereby one sets the model’s boundary by determining the input and output, in addition to giving a systemic view of the process interactions between subcomponents.

The flow chart offers a simple overview of the whole patient flow process and provides valuable direct guidance on how to construct the statechart of the following computational model.

Lastly, in the conceptual modeling process, the sequence diagram captured and highlighted the feedback loops and the plethora of different realizations throughout the patient flow system that the ED entails. In particular, the sequence diagram highlighted how much the complexity of the patient flow increased when the ED had to perform the prioritization of regular and contamination suspected patients.

In their unique way, each of these tools helped arrive at the necessary understanding of the patient flow complexities to proceed to the computational modeling. We assert that this understanding goes beyond the understanding one can gain by solely using a flowchart as a conceptualizing tool, as commonly seen in many patient-flow simulation studies.

Utilizing the conceptual modeling approach as demonstrated in this study is useful as it demonstrates the use of a set of tools for data collection and communication with the stakeholders. It can also be used to verify and validate if the conceptual model represents a credible understanding of the real-world case and to validate if the computational model is traceable to the conceptual model. It is important to emphasize the benefit of the tools being software independent.

The synergic effect of the combination of the different tools is essential. The tools represent four abstractions of the same patient flow process. They serve as four different low-resolution lenses to perceive the actual process. We believe that the diversity within these “lenses” forces the simulation modeler and the participating stakeholders to perceive the system from different conceptual perspectives. This, in turn, helps extract as much relevant information as possible, in line with the stated purpose(s) of a conceptual model, thus increasing the chance of representing the actual system behavior. We believe the approach we have followed helps the stakeholders articulate important elements they otherwise might consider too obvious to mention or that they may have not reflected thoroughly upon.

### 4.2. Computational Model

The computational model, represented by a discrete event process diagram and statecharts, has been structured based on the conceptual model diagrams. It is clear that any computational model has a structure, behavior (formulas, rules, etc.) and inputs. The rules and conditions have been extracted from interviews with staff working and managing the ED at SUS. The input data such as ‘time of arrival’ was extracted from a real-time database, i.e., Meona. The service time for treatment and waiting time are assumed based on expert experience. Finally, the simulated results were validated in two manners: (1) compared with real-life patient flow data (2) dialogue with stakeholders in the ED to ensure that our understanding has mimicked real-world structure and relationships.

The comparison between the actual real-life data provided by SmartCrowding and the simulation results provided by our model, illustrated in Table 3, clearly shows similarities in the daily pattern (i.e., curve shape) of the patient prevalence in the ED. Producing a realistic output that reasonably replicates the system behavior, confirmed by the expert group in the meetings as being credible, gained confidence from the group. We experienced that this confirmation was a sign of trustworthiness of the model and yielded our acceptance with the system stakeholders as the model continued to develop.

One particular problem experienced during the model development, which had a practical solution, was the way to emulate the limited resources (i.e., emergency treatment rooms). This was solved by making these model elements use discrete-event modeling rather than agent-based modeling. There were, thus, interfaces between the DES portion of the model and the ABM part of the model. Agents would be programmed to temporarily exit their statechart, seize their supposed resources, and return to progress through the statechart.

The resulting model ended up fairly complex. Data will be gathered and calculated every second, and the agents will check conditions for several transitions. However, a regular modern laptop can run this model, simulating 24 h of operation in less than one minute.

The computational model provides reliable and consistent results in several runs. However, three assumptions have been taken regarding the model: (1) static patient infection rate, (2) no intra-hospital contamination, and (3) perfect sorting of the patients in the pre-screening. The patient rate is modeled as a variable that users of the model will have the ability to vary. However, intra-hospital contamination and imperfect sorting might require further development to model such complex phenomena. It is worth highlighting that the developed simulation model is generalizable in two terms. First, it can be utilized for other EDs in Norway or hospitals with similar operating procedures. The process of ED patient flow in our case organization widely coincides with the commonly used process described in the ED report from the Norwegian Directorate of Health [27]. Second, it has the potential to be utilized for other pandemics with different types of diseases.

The resulting computational model constitutes perhaps the biggest contribution of this paper. Being a hybrid model, including two major simulation paradigms: agent-based simulation and discrete event simulation, it is a result consistent with the recommendation of other studies to use more advanced simulation methods to incorporate more of the underlying complexities of the healthcare delivery system [10].

#### 4.2.1. Simulation Model Validity

The conceptual model, represented by the purpose tree, N2-matrix flowchart, and sequence diagram, has been extracted from discussions with staff working at and managing the ED at SUS. Through continual and incremental verification from the system experts, the model was developed and tailored to mimic the patient flow system of the entire ED in the case hospital.

Confidence in the computational model was achieved by running the model and comparing the simulation output with actual data, both in a pre-pandemic and a peri-pandemic setting. There were sufficient overall similarities between real-world data and simulated data in both settings to gain confidence in the model’s accuracy.

Our simulation model does not yet provide point prediction [28] in the sense that the simulation results yielded the same results at every time point as the empirical data from the same period. As mentioned, the simulation output from our computational model (Table 3, row b) showed some discrepancies in the empirical data (Table 3, row a) that covered the same periods (2 separate days) as our simulations. The discrepancies between (a) the real and (b) the simulation output data were subject to discussion with the case organization stakeholders and possible explanations were offered.

One explanation could be our simplified assumption of a 2,0-hour treatment time for every patient. Although our participating stakeholders at the case hospital suggested this simplification, they did indicate that treatment times would vary in practice. In particular, they stated that under periods of high patient crowding, most procedures tend to take longer than average, thereby extending the treatment time. Under these conditions, patients will stay in the system longer than the current simulation model assumes, thereby elevating the empirical curves higher than the simulation curves, as shown in Table 3. Our stakeholders based this perception (calling it the “syrup effect”) on their experience rather than any available empirical data. We intend to develop our simulation model to address this issue in our future simulation studies. However, given the underlying assumptions and simplifications necessary in any simulation model, we assert that our model provides a reasonably good pattern prediction [28].

Another explanation offered by the stakeholder group was the subtle difference present in reporting between the two datasets. The real data gathered from the SmartCrowding system, plotted in row ‘a’ of Table 3, tracks the number of patients registered to the ED. This means that patients traveling to the hospital from their general practitioners or via an ambulance are registered as prevalent patients. However, the data used in the simulation model has a registration based upon actual arrival in the physical ED space. This difference results in a delay between the curves, as observed in Table 3, where the simulation output is slightly lagging behind the real data. This discrepancy will also be taken into consideration in future model development.

The differences between our simulation output and the actual patient flow data might also be explained by other phenomena that take an active role in the ED, such as the latency associated with higher crowding, staff shifts, or higher variation in the treatment times. The differences might also highlight the implication of the model delimitations that we have taken. For example, patients and rooms were the only two modeled agents; medical staff (doctors, nurses) and beds were excluded. Moreover, the interactions between the ED and other departments were excluded, such as blood test, and X-ray laboratories, where patients are usually sent to those departments between their stay at the ED.

The “syrup effect,” potential delays in availability of doctors, nurses, and beds, and time spent at other departments all directly affect patient flow in the ED. In fact, such issues show the need and implication of using a multi-method simulation approach instead of only a discrete event or system dynamics approach. In future model development, more agents shall be considered in order to mimic the real-world behavior of the ED.

### 4.3. Assumptions and Simplifications

In addition to the above, there are other assumptions and simplifications that were taken at this stage of model development, as follows:

1. The percentage of COVID-19-contaminated patients out of total patients entering the ED, i.e., the patient contamination rate (PCR), was considered 30% in the peri-pandemic scenarios and 0% in the pre-pandemic scenarios. This percentage was constant throughout the entire day, while it may have varied throughout the day in real life.
2. Treatment time for each patient agent was assumed to be constant. However, realistically, the treatment time will most likely vary throughout the day according to what types of patients arrive to the emergency department. In this model, the treatment time was set static to a 2-hour average, based on an assessment by the case stakeholders.
3. No intra-hospital contamination was put into the model.
4. Therefore, the model implicitly assumes that there is a perfect sorting, i.e., a sorting error is not taken into consideration regarding the patients in the pre-triage.

### 4.4. Practical Implications

This work was carried out in the context of the COVID-19 pandemic. However, the model we have developed can be applied to other pandemic contexts, provided they require similar patient flow operation and intervention policies similar the ones used in this paper, i.e., that infected patients needs to go directly to their own treatment room and should avoid the waiting room and triage. On the other hand, the transparency of our account should facilitate other researchers to make necessary modifications to the model to suit their particular contexts.

Although the interventions (i.e., pre-triage, waiting zone) might be unique for this particular ED in our study’s context, this paper shows how such interventions can be brought into a computational model.

The interventions shown here are a snapshot of one particular time in the case organization. Such policies will need to change in accordance with the best medical knowledge at the time in order to maintain the required quality of treatment. Detailed modeling can be quite time-consuming, and in the worst case, a model can be deemed obsolete by the time it is ready to be used. Additionally, it might be difficult for researchers to gain access to relevant stakeholders in such a high-paced and hands-on environment as an ED. There will likely be a need and demand from these to achieve fast-paced tangible outcomes to maintain stakeholder interest.

### 4.5. Further Work

As the scope of this paper was only to show the model building itself, a natural continuation from this is to carry out actual simulation tests. A natural expansion of the work presented in this paper is to progress further onto the next step in Randers’ overall simulation modeling process, as illustrated in Figure 3. In subsequent work, we will demonstrate how our simulation model can be used to study the effects of pandemic-related policies on patient flow. For example, introducing a waiting zone and/or an extra treatment room in the ED are some of these policies.

Although the model resulting from the study presented in this paper is relatively advanced compared to previous studies, since multiple agents and multi-method simulation approaches are considered, we believe this study is merely scratching the surface of what truly is the potential of ABM in conveying patient flow problematics. The proposed model can be expanded in several different directions. In this regard, there are several avenues for further work in the modeling work presented in this study. Examples are:

- Replacing our assumption of a constant contamination rate with a dynamically changing rate as a pandemic develops through time throughout a population.
- Replacing our assumption of no intra-hospital contamination with a probability of such contamination.
- Replacing our assumption of perfect sorting of incoming patients with a probability of classification inaccuracies.

## 5. Conclusions

This study set out to model the patient flow behavior in the ED under pandemic conditions. We conclude, based on a comparison between actual and simulated behavior of patients, that patient flow behavior under pandemic conditions with multiple agents (patients, department resources) and operational complexities (transitions, conditions, priorities, processing time, resource limitations) can be modeled with an acceptable degree of accuracy. We found that the prioritization of COVID-19-contaminated patients has complicated the ED behavior and has significantly affected the studied key performance measures. Moreover, compared to the actual data, the simulated results highlight the need for further study, including other vital agents (doctors, nurses, beds, other departments) and behavioral phenomena related to the studied patient flow regiment.

The final simulation model could emulate the patient flow behavior, as this research set out to accomplish. This was confirmed with stakeholder informants who had extensive knowledge and familiarity with the system itself. By this, the model was confirmed to pose a valid re-representation of the patient flow process of the case organization.

We found and concluded that neither discrete-event nor agent-based modeling on their own was effectively able to encompass the resulting patient flow complexities stemming from the COVID-19 pandemic. However, combining the two in a hybrid simulation model capitalized on the strengths from both discrete-event and agent-based modeling. The strength utilized from discrete-event modeling was the ability to model the resource allocation between patient agents. Similarly, the strength utilized from agent-based modeling was the ability to model the patient agent’s complex journey through the system.

In line with our stated research goals, this paper has provided:

- A conceptual and a computational model that mimics, explains, and predicts ED behavior under pandemic conditions while considering multiple agent behaviors.
- An account demonstrating the rigorous and transparent implementation of conceptual modeling of patient flow in the ED, to a greater extent than shown in previous research literature. By documenting the entire thought process behind each step of the modeling process and showing the resulting model, we believe that we contribute to modeling practice by making these steps transparent and accessible to others. This will be helpful to other researchers to understand, assess and build trust in such models, and provides an example than can be emulated by others to build their own thoroughly validated models.
- The computational model we have provided includes several performance measures that will be useful for practitioners but have not been previously introduced in simulation studies of patient flow in the ED.
- Furthermore, we have discussed the broader applicability of our models and are currently undertaking further research using the presented model to study patient flow in the ED under pandemic conditions.

## Figures and Tables

**Figure 14:**
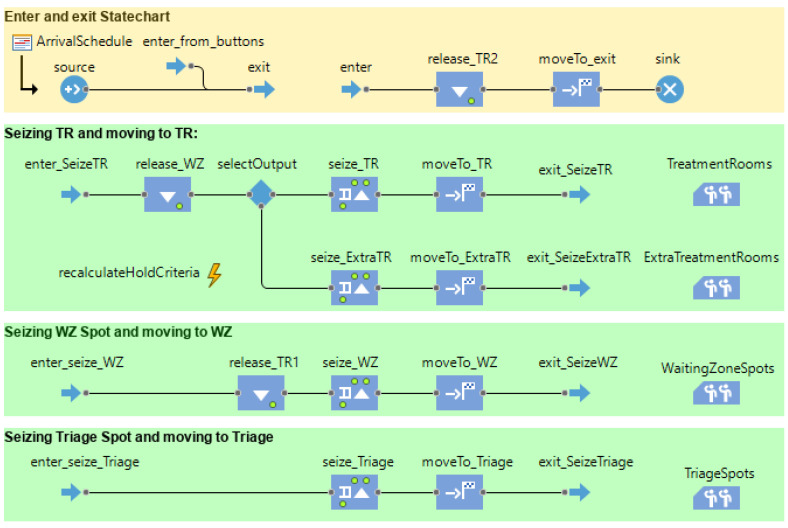
Process diagram programmatically carrying out the resource allocation. Patients are input in the source and output in the sink; resources will be seized and released depending on the individual patient agent’s situation.

**Table 1:** **a.** Patient crowding in the ED gathered from Smart Crowding for two regular days in a pre-pandemic situation. **b.** Patient crowding in the ED gathered from Smart Crowding for two separate days in a peri-pandemic situation.

a		
	Day 1	Day 2 ^†^
Real data	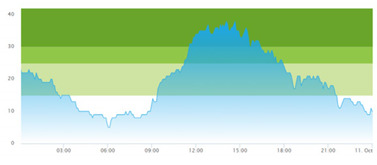	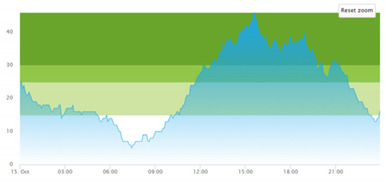
**b**		
	**Day 1**	**Day 2 ^†^**
Real data	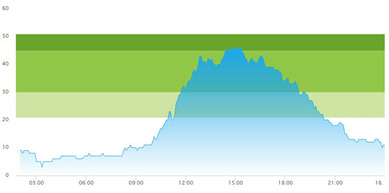	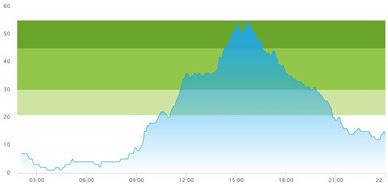

† Graph “Day 2” is not immediately the following day after “Day 1” as the naming may suggest.

**Table 3:** **a.** Model output validation comparing graphs of patient crowding in the ED in a pre-pandemic situation. (**a**) Actual patient crowding data gathered from SmartCrowding, (**b**) Simulated patient crowding. **b.** Model output validation comparing graphs of patient crowding in the ED in a peri-pandemic situation. (**a**) Actual patient crowding data gathered from SmartCrowding, (**b**) Simulated patient crowding.

a		
	Day 1	Day 2 ^†^
(**a**) Actual patient crowding	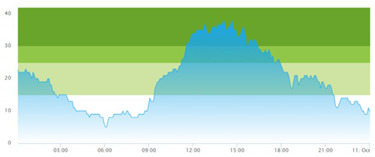	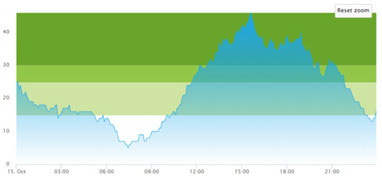
(**b**) Simulated patient crowding	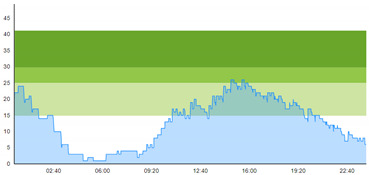	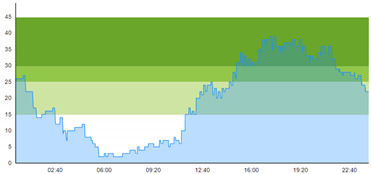
**b**		
	**Day 1**	**Day 2 ^†^**
(**a**) Actual patient crowding	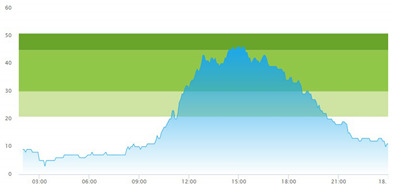	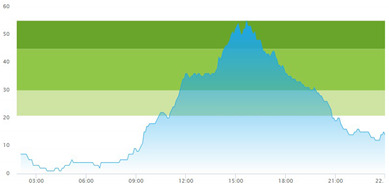
(**b**) Simulated patient crowding	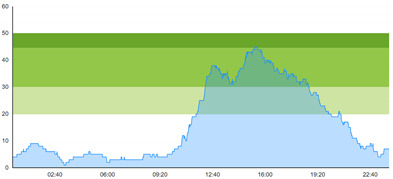	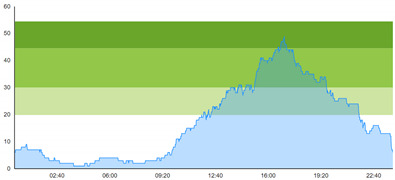

† Graph “Day 2” is not the immediately following day after “Day 1” as the naming may suggest.

## Data Availability

The numerical data used in this study is not publicly available and thus is not for distribution.

## Abbreviations

**ABM**—Agent-based modeling, **ED**—Emergency departments, **PF**—Patient flow, **SUS**—Stavanger Universitetssykehus (The University Hospital of Stavanger), **WZ**—Waiting zone, **TR**—Treatment room.
